# Sundew plant, a potential source of anti-inflammatory agents, selectively induces G2/M arrest and apoptosis in MCF-7 cells through upregulation of p53 and Bax/Bcl-2 ratio

**DOI:** 10.1038/cddiscovery.2015.62

**Published:** 2016-01-18

**Authors:** NB Ghate, A Das, D Chaudhuri, S Panja, N Mandal

**Affiliations:** 1 Division of Molecular Medicine, Bose Institute, P-1/12 C. I. T. Scheme VII M, Kolkata 700054, India

## Abstract

The worldwide cancer incidences are remarkable despite the advancement in cancer drug discovery field, highlighting the need for new therapies focusing on cancer cell and its microenvironment, including inflammation. Several species of Drosera (family: Droseraceae) are used in various traditional as well as homeopathic systems of medicine. *Drosera burmannii* Vahl. is also enlisted in *French Pharmacopoeia* in 1965 for the treatment of inflammatory diseases, including chronic bronchitis, asthma and whooping cough. The present study is designed to substantiate the potential of *D. burmannii* in *in vitro* anticancer activity and its relation with anti-inflammatory property. *In vitro* anticancer study revealed that DBME is inhibiting the proliferation of MCF-7 cells without affecting the viability of other malignant and non-malignant cells. DBME induced G2/M phase arrest and apoptosis in MCF-7 cells by suppressing the expression of cyclin A1, cyclin B1 and Cdk-1 and increasing the expression of p53, Bax/Bcl-2 ratio leading to activation of caspases and PARP degradation. Presence of caspase-8 (Z-IETD-fmk) and caspase-9 (Z-LEHD-fmk) inhibitors alone did prevent the apoptosis partially while apoptosis prevention was significantly observed when used in combination, suggesting vital role of caspases in DBME-induced apoptosis in MCF-7 cells. DBME also downregulated LPS-induced increased expression of iNOS, COX-2 and TNF-*α* along with suppression on intracellular ROS production that confirms the potential of DBME as anti-inflammatory extract. GCMS analysis revealed the presence of four major compounds hexadecanoic acid, tetradecanoic acid, hexadecen-1-ol, trans-9 and 1-tetradecanol along with some other fatty acid derivatives and carotenoids (Beta-doradecin) in DBME. These findings confirmed the anti-inflammatory activity of DBME, which is already listed in *French Pharmacopeia* in 1965. Here we have additionally reported the anti-breast cancer activity of DBME and its relation to the anti-inflammatory potential. Hence, an ethnopharmacological approach can be considered as useful tool for the discovery of new drug leads.

## Introduction

The emergence of cancer is a complex multistep process during which normal cells progressively acquire abnormal growth leading to cancer. The role of inflammation in cancer initiation and progression is well known and the relevant molecular mechanisms have been dissected widely. Therefore, in the past few decades, it has become practically possible to target inflammatory pathways for cancer prevention and therapy. Inflammation can be considered as a central feature of many pathophysiological conditions that are initiated in response to tissue damage and infection and leads to the secretion of cytokines and other mediators as well as activation and migration of immune cells. These cytokines/mediators add to the generation of excess free radicals such as reactive oxygen species (ROS) and reactive nitrogen species (RNS), which leads to DNA damage, mutilation of DNA-repair proteins and caspases and lipid peroxidation, followed by mutation and NF-*κ*B activation; all these phenomena give rise to broad range of diseases, including cancer.^1^ In spite of the development in the field of anticancer drug discovery, the statistics are disappointing; in 2012, 14.1 million new cases of cancer were diagnosed worldwide, with 8.2 million deaths.^2^ Thus there is still a necessity for the advancement of new therapies, and the tumor microenvironment, including inflammation, can be considered as a source of multiple targets for cancer therapy.^3^ Moreover, evidence shows that inflammation not only contributes to cancer development but also affects the effectiveness of chemotherapy.^4^ Hence, the search for efficient anticancer agents of natural origin, which not only treats cancer but also contributes in prevention through anti-inflammatory activity, is the need of the hour.

*Drosera burmannii* Vahl. is an insectivorous herb commonly known as sundew and belongs to the family Droseraceae, one of the largest genuses of carnivorous plants with >105 species. It is distributed throughout northern and eastern Australia, India, China, Japan and South-east Asia. Different types of *Drosera* sp. have been traditionally used in Europe to treat several inflammatory diseases, including chronic bronchitis, asthma and whooping cough, and also were listed in *French Pharmacopeia* in 1965. In 1880, Murray described its various uses in the ‘Royal Hospital of a whooping cough’.^5^ Alcohol and aqueous extracts of *D. burmannii* act as anticonvulsants^6^ and possess antitumor activities on mice.^7^ Our previous studies have established the role of *D. burmannii* as an antioxidant and *in vivo* alleviation of iron overload induced hepatotoxicity.^8^ However, there has been no report on the anti-inflammatory as well as an anticancer properties of this species. Keeping these in mind, the need for new therapies focusing on the tumor microenvironment and the potential of *D. burmannii* as an anticancer agent non-toxic to the non-malignant cells, in the present study the *in vitro* antiproliferative activities of its 70% methanolic extract (DBME) was evaluated. To connect shreds of evidences describing the relationship between anticancer and anti-inflammatory activities of DBME, the latter was also examined along with gas chromatographic investigation of DBME.

## Results and Discussion

### In vitro anticancer study

#### DBME inhibits cell proliferation of breast cancer MCF-7 cells

**Figure 1:**
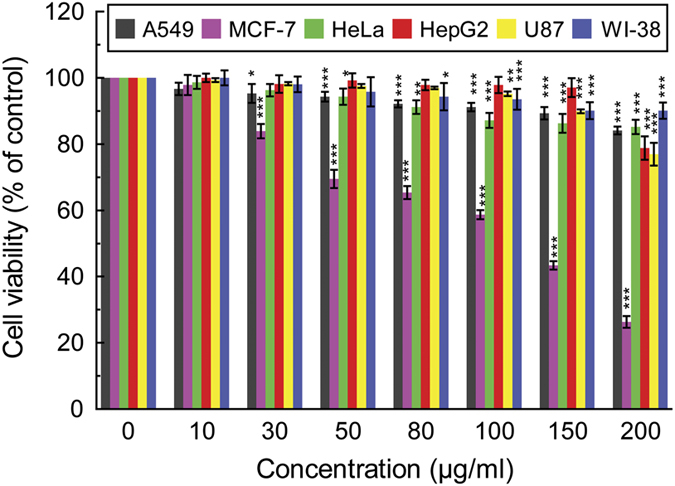
Effects of DBME on cell proliferation and viability of A549, MCF-7, HeLa, HepG2, U87 and WI-38 cells. Cells were cultured in 96-well plates and treated with the indicated concentrations of DBME (0–200 *μ*g/ml) for 48 h. Cell proliferation and viability was determined with WST-1 assay. Data are expressed as mean±S.D. (*N*=6). *P<0.05, **P<0.01 and ***P<0.001 *versus* 0 *μ*g/ml.

The cytotoxicity of DBME on lung (A549), breast (MCF-7), cervical (HeLa), liver (HepG2) and brain (U87) cancer cells along with normal fibroblast cell line (WI-38) was performed; the results are shown in Figure 1. Comparing the results of untreated (control) and treated groups, in case of MCF-7 cells, treated samples exhibited a dose-dependent decline in viability, so that the highest reduction in viability was rendered to 25.89% by 200 *μ*g/ml of the extract after 48 h of incubation and the IC_50_ value was found to be 120.94±1.91 *μ*g/ml. However, no significant suppression was found in cell proliferation of other cancer and WI-38 cells after the treatment of DBME as corroborated by their high IC_50_ values (Table 1). This result indicates that DBME has selective growth-inhibitory activity against breast carcinoma (MCF-7) but not to the other cancer cells while being non-toxic to the normal fibroblast cells (WI-38).

#### DBME induces G2/M phase arrest and apoptosis in MCF-7 cells

**Figure 2:**
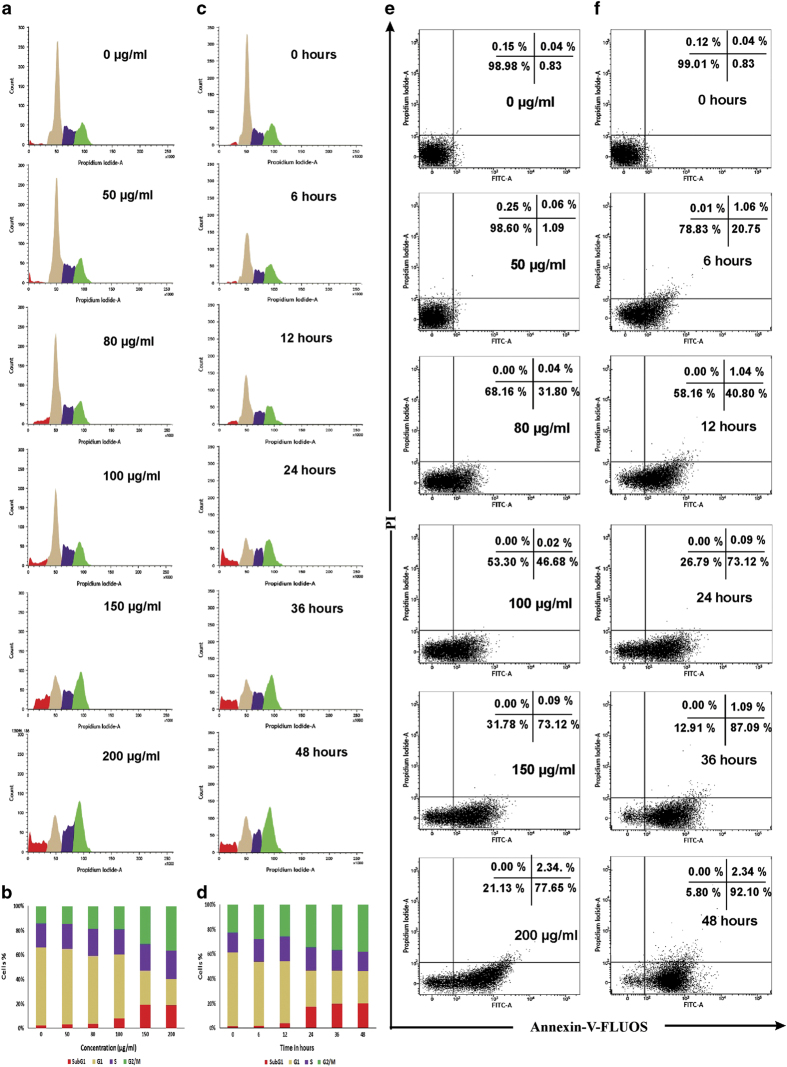
Flow cytometric analysis of MCF-7 cells treated with DBME. (**a**) Cell cycle phase distribution of MCF-7 cells treated with the indicated doses for 48 h. (**b**) Graphical representation of the percentage of cell population in Sub-G1, G1, S and G2/M phases for panel (**a**). (**c**) Cell cycle phase distribution of MCF-7 cells treated with DBME (200 *μ*g/ml) for the indicated time intervals. (**d**) Graphical representation of the percentage of cell population in Sub-G1, G1, S and G2/M phases for panel (**b**). (**e**) Apoptosis detection of MCF-7 cells treated with the indicated doses for 48 h. (**f**) Apoptosis detection of MCF-7 cells treated with DBME (200 *μ*g/ml) for the indicated time intervals.

It is reported that some anticancer agents directly induce apoptosis or arrest the cells at G0/G1, S and G2/M phases of cell cycle and then induce apoptosis^9,10^ to kill cancer cell.^11^ The effect of DBME on cell cycle distribution of MCF-7 cells was studied to verify the mechanism by which growth-inhibitory effect was achieved. Results show that DBME arrested MCF-7 cells in G2/M phase in a dose- as well as time-dependent manner and also induced apoptosis as confirmed by increase in the sub-G1 population. DBME treatment leads to the accumulation of cells in G2/M phase from 14% (0 *μ*g/ml) to 36.57% (200 *μ*g/ml) and increase in sub-G1 population from 3 to 18%. Similar cell phase distribution was also observed when cells were treated at different time intervals (Figures 2a–d). Moreover, Annexin-V-staining study also depicted that DBME induced dose-dependent induction of apoptosis in MCF-7 cells (Figure 2e). The results showed an upsurge in early apoptosis with the increasing doses of DBME. At zero dose, 0.83% cells were found in early apoptotic phase, which was increased to 1.09, 31.80, 46.68, 73.12 and 77.65% with 50, 80, 100, 150 and 200 *μ*g/ml doses, respectively, suggesting that DBME kills MCF-7 cells through apoptosis and not through necrosis. In all, 20.75% cells in 6 h, 40.80% in 12 h, 73.12% in 24 h, 87.09% in 36 h and 92.10% in 48 h were found to be in early apoptotic stage (Figure 2f). The results from time- as well as dose-dependent flow cytometric study indicate that DBME is effective in inducing G2/M phase arrest as well as apoptosis in MCF-7 cells, suggesting that further investigations are needed to clarify the mechanism of arrest and apoptosis.

#### DBME regulates the expression of cell cycle regulatory proteins and activates tumor-suppressor p53 in MCF-7 cells

**Figure 3:**
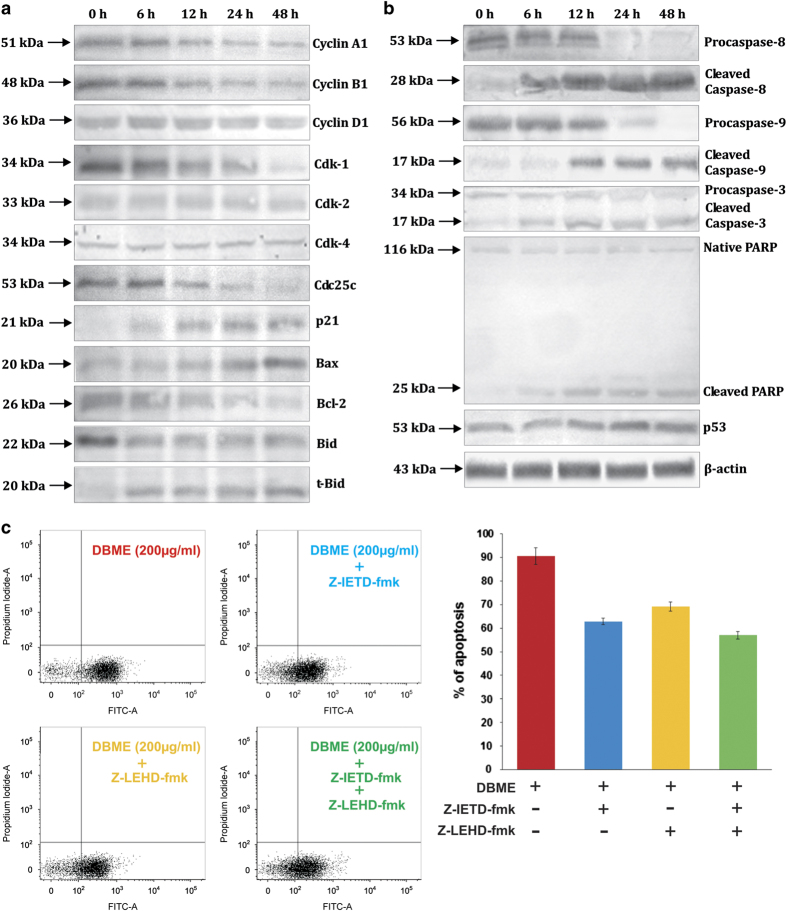
Immunoblot analysis of cell cycle regulatory and apoptosis-related proteins of cell lysates of MCF-7 cells treated with DBME (200 *μ*g/ml) for the indicated time intervals. (**a**) Cell cycle, Bcl-2 family, p21 proteins. (**b**) Caspase family, PARP, p53 proteins. (**c**) Effects of caspase inhibitors on DBME-induced apoptosis in MCF-7 cells. The cells were treated with Z-IETD-fmk, Z-LEHD-fmk and both in the presence of DBME (200 *μ*g/ml) for 48 h and analyzed by Annexin-V–PI staining using flow cytometer. Data are expressed as mean±S.D. (*N*=3).

The cell cycle is a controlled process and possesses checkpoints so that the DNA-damaged cells get repaired and enter into the next phase. The G2/M checkpoint is a potential target for anticancer drugs of chemotherapy. This checkpoint prevents the entry of DNA-damaged cells from entering mitosis and allows repairing of the DNA that was damaged in late S or G2 phases.^12^ It is known that cyclin A1/Cdk-1 and cyclin B1/Cdk-1 complexes are important for the transition from G2 phase to M phase.^13^ Therefore, we examined the effect of DBME on the expression levels of various cell cycle regulatory proteins (Figure 3). Western blotting results revealed that the expression levels of cyclin A1, cyclin B1 and Cdk-1 was decreased dramatically with increase in time after DBME treatment. However, Cdk-2 did not get affected by the treatment. Cdc25C dephosphorylates Cdk-1, facilitating cyclin/Cdk complex formation, which is required by the cell for entry into mitosis that is needed for transition of G2–M phase. The expression of Cdc25C was also downregulated time-dependently after the treatment.^14^ Alongside, the complex of cyclin D1 and Cdk-4 is responsible for the transition of cell cycle from G1 phase to S phase; their immunoblot analysis depicts no change in their protein expression levels after the treatment of DBME, which suggests that DBME has no role in G1-phase regulation. p53 is a tumor-suppressor protein and has essential regulatory mechanism in cell cycle and apoptosis. In some cases, activation of p53 can be caused by the stresses such as DNA damage; resulting in the cell cycle arrest at G0/G1, S or G2/M phase by activating its downstream Cdk inhibitor p21 or by inhibiting cyclins and Cdks directly.^15,16^ The present study showed a significant induction in the expression levels of both the proteins. These results suggest that decrease in the expression of necessary cyclins and Cdks and increase in p53 and p21 expression levels may be the molecular mechanism through which DBME induced G2/M arrest in MCF-7 cells.

#### DBME increases Bax/Bcl-2 ratio and activates caspase cascade in MCF-7 cells

Caspases are the members of cysteine proteases family that are expressed as inactive enzymes and have a vital role in apoptosis.^17^ Procaspase-9 responds to the release of cytochrome *C* from mitochondria and interacts with Apaf-1 and gets activated to caspase-9, resulting in the activation of caspase-3. This activated caspase-3 proteolytically degrades PARP, completing the intrinsic pathway of apoptosis. In the case of extrinsic pathway, death-receptor-mediated active caspase-8 directly activates downstream executioner caspase-3 by proteolytic cleavage.^18^ To determine the mechanism of DBME-induced apoptosis in MCF-7 cells, the expression of pro-apoptotic and antiapoptotic proteins following DBME treatment was also examined using western blotting (Figure 3). It was found that pro-caspase-9, and -3 gets downregulated, which results in the increasing levels of cleaved caspase-9 and -3, and degradation of PARP to its cleaved form. The balance between Bax (pro-apoptotic) and Bcl-2 (antiapoptotic) expression levels is critical for cell survival and death. It was reported that increase in Bax/Bcl-2 ratio contributes to the activation of intrinsic apoptotic pathway through release of cytochrome *C* from mitochondria.^19^ DBME treatment also leads to the time-dependent increase in Bax/Bcl-2 ratio as a consequence of upregulation of Bax and downregulation of Bcl-2, contributing to DBME-induced apoptosis in MCF-7 cells. The level of cleaved caspase-8 is elevated, whereas that of procaspase-8 is decreased; simultaneously t-Bid also appeared after DBME treatment. Activated caspase-8 links the extrinsic and intrinsic pathways by cleaving Bid into its truncated form (t-Bid), thus contributing in activation of both the pathways of apoptosis.^20^ These results suggest that DBME induces apoptosis in MCF-7 cells via upregulation of Bax/Bcl-2 ratio and activation of caspases.

#### DBME activates intrinsic and extrinsic pathways, independently

The aforementioned results suggest that DBME activates both extrinsic and intrinsic pathways of apoptosis in MCF-7 cells. Both the pathways converge and involve activation of effector caspase (caspase-3) but possess two different initiator caspases (caspase-8 and -9) as mentioned earlier. To address whether or not caspase-8 activation is preceded by caspase-9 or vice versa, cells were preincubated with Z-IETD-fmk (caspase-8 inhibitor) or Z-LEHD-fmk (caspase-9 inhibitor) or combination of both, before the treatment of DBME. After treatment, DBME-induced apoptosis was investigated using Annexin-V/PI staining, and the obtained results are shown in Figure 3c. It was found that caspase-8 inhibitor decreases DBME-induced apoptosis from 91 to 61% and caspase-9 inhibitor from 91 to 67%; however, the presence of both the inhibitors leads to decrease in apoptotic cells from 91 to 56%. As the inhibition of Z-LEHD-fmk (1.3-fold) is lesser than Z-IETD-fmk (1.5-fold), we suggest that caspase-8 activation may be a major apoptotic signaling pathway. Conversely, caspase-8 inhibitor alone was not able to completely inhibit apoptosis, indicating that caspase-9 is also getting activated independently. Similarly, caspase-9 inhibitor alone induces partial inhibition of apoptosis, indicating that caspase-8 also directly targets caspase-3 promoting apoptosis independently. Appearance of Bid truncation may be considered as a subsidiary event contributing to the activation of intrinsic pathway by DBME. These results suggest that both extrinsic and intrinsic pathways are having a causal role in apoptosis triggered due to DBME. Additionally, when both the inhibitors were used in combination, an effective inhibition (1.7-fold) of apoptosis was observed. This inhibition was better as compared with those caused by the inhibitors alone. However, the combination of both the inhibitors also failed to completely inhibit apoptosis. As DBME is a crude extract, many different phytochemicals may act differently, and hence, several other targets might also be associated with DBME-induced apoptosis in MCF-7 cells. Additionally, it was previously reported that the crude extract also induce apoptosis through senescence followed by G1 block.^21^ Surprisingly, several studies reported that caspase activation blockade, by caspase inhibitor, lead to autophagic cell death.^22,23^ Therefore, these results warrants further in-depth investigations of each of the bioactive compounds present in DBME for determination of target involved in anticancer activity against MCF-7 cells.

### In vitro anti-inflammatory study

#### DBME decreases LPS-induced NO, TNF-*α* and ROS production in RAW 264.7 cells

**Figure 4:**
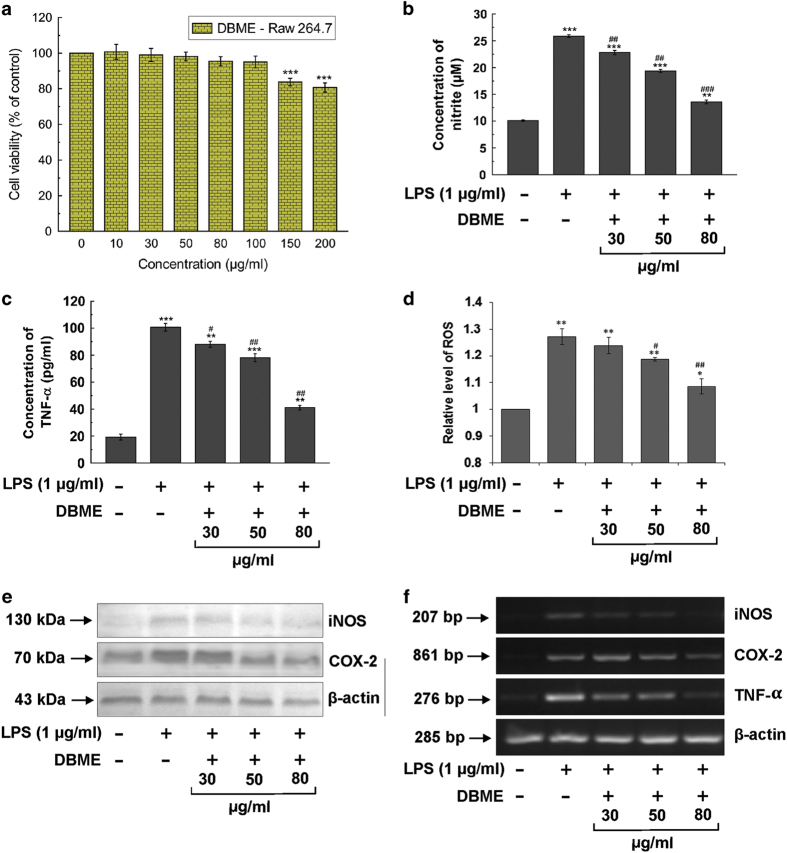
*In vitro* anti-inflammatory effects of DBME in RAW 264.7 cells. (**a**) Effect of DBME on cell proliferation and viability of murine macrophage RAW 264.7 cells. (**b**) Effect of DBME on LPS-induced production of nitrite. (**c**) Effect of DBME on LPS-induced production of TNF-*α*. (**d**) Effect of DBME on LPS-induced production of ROS. (**e**) Effect of DBME on LPS-induced expression of inflammatory proteins. (**f**) Effect of DBME on LPS-induced mRNA levels of inflammatory proteins in RAW 264.7 cells. Cytotoxicity data are expressed as mean±S.D. (*N*=6); *P<0.05, **P<0.01 and ***P<0.001 *versus* 0 *μ*g/ml. Nitrite, TNF-*α* and ROS data are expressed as mean±S.D. (N=3); *P<0.05, **P<0.01 and ***P<0.001 *versus* 0 *μ*g/ml LPS. ^#^*P*<0.05, ^##^*P*<0.01 and ^###^*P*<0.001 *versus* 1 *μ*g/ml LPS.

Before determining the effect of DBME on LPS-induced NO, TNF-*α* and ROS production in RAW 264.7, the cytotoxicity of DBME on RAW 264.7 cells was examined using the WST-1 reagent. The cytotoxic effect was tested to establish the appropriate concentration ranges of DBME for the analysis of ongoing experiments (Figure 4a). The non-toxic concentrations (30, 50 and 80 *μ*g/ml) were selected for further experiments. These concentrations significantly inhibited the LPS-induced nitrite (Figure 4b), TNF-*α* (Figure 4c) and ROS (Figure 4d) production in RAW 264.7 cells. After 24 h, LPS produced a considerable increase in NO and TNF-*α* in culture media and intracellular ROS. DBME drastically inhibited nitrite, TNF-*α* and ROS production in a dose-dependent manner, with a maximum effect at 80 *μ*g/ml.

#### DBME downregulated LPS-induced protein and mRNA expression levels of iNOS and COX-2 in RAW 264.7 cells

As DBME promisingly decreased the production of nitrite in LPS-stimulated RAW 264.7 cells, we next examined the effect of DBME on the protein expression of NO-synthesizing enzyme iNOS. The immunoblot results suggest that DBME significantly downregulated the protein expression of iNOS in a dose-dependent manner. In case of COX-2, a drastic downregulation of its expression has been observed after treatment with different concentrations (Figure 4e). As a whole, DBME displayed a remarkable inhibitory effect on LPS-induced enhanced protein expression levels of iNOS and COX-2; therefore, mRNA expression levels of these two proteins along with TNF-*α* have been further investigated. DBME significantly inhibited the mRNA expression levels of iNOS, COX-2 and TNF-*α* (Figure 4f), which suggested that the suppressive effect of DBME may be primarily through transcriptional mechanisms. Although the mechanism of tumor development involves distinct etiologic factors, local persistent tissue inflammation is commonly involved in carcinogenesis. Several studies also demonstrated the involvement of iNOS in cancer progression and angiogenesis.^24,25^ COX-2 has a key tumor-promoting role, and its expression is observed early during the tumorigenesis. It is reported that celecoxib, a COX-2 inhibitor, decreases the proliferation and increases the apoptosis in tumor and stromal cells.^26^ On the other hand, TNF-*α* is involved in the promotion and progression of experimental and human cancers through activation of NF-*κ*B. TNF-*α*, in high doses, causes necrosis via selective destruction of tumor blood vessels and generation of specific T-cell antitumor immunity,^27^ but, when produced in tumor microenvironment, leads to the endogenous tumor promotion.^28^ As there is a causal relationship between inflammation and cancer, iNOS, COX-2 and TNF-*α* are often considered as potential molecular targets for chemoprevention.

### GCMS analysis of DBME

**Figure 5:**
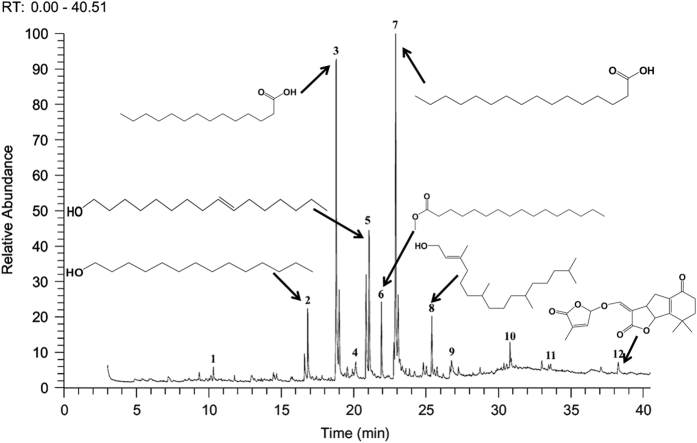
GCMS chromatogram of 70% methanolic extracts of *D. burmannii*. The numbers 1–12 represents considerable peaks in the chromatogram and the identified compounds listed in Table 2.

It is well recognized that the medicinal properties of plants are largely attributed to the phytochemicals present in them. Our previous study indicates that DBME is a potential source of phenolics, flavonoids, alkaloids, carbohydrates, tannins and ascorbic acid. However, HPLC analysis depicted the presence of purpurin, catechin, tannic acid, reserpine, methyl gallate and rutin. Moreover, when the active components in DBME identified by HPLC were tested individually for their toxicity toward normal cells (WI-38), it was found that most of the identified compounds excluding methyl gallate and reserpine were non-toxic to normal cells.^8^ The GCMS chromatogram of the extract (DBME) (Figure 5) consists of 12 peaks, which have been identified by comparing the retention times as well as the spectral data with different phyto-compounds obtained from the library. The individual names of the compounds and their structures identified with respect to their individual peak value with their peak number, retention time and the percentage of area are listed in detail in Table 2. Among these, hexadecanoic acid was present in 24% (peak no. 7), forming one of the major constituents in the extract followed by 21% of tetradecanoic acid (peak no. 3), 15% of hexadecen-1-ol, trans-9 (peak no. 5) and 6% of 1-tetradecanol (peak no. 2). Some of the minor constituents are methyl palmitate (3.98%), 2-hexadecen-1-ol, 3,7,11,15-tetramethyl (3%), octadecanoic acid (3.11%) and 2-(hexadecyloxy) ethanol (3.15%). All detected constituents are fatty acids or derivatives of fatty acids; moreover, minute quantities of a carotenoid, Beta-doradecin (1.26%), were also found.

Carotenoids are well known for their potent and synergistic effect against several oxidative stresses.^29^ The major constituent of DBME, hexadecanoic acid (palmitic acid), was reported for its anti-inflammatory activity^30^ and selective cytotoxicity against human leukemic cells.^31^ The other major component, that is, 1-teradecanol was reported to be useful in the treatment of periodontitis, an inflammatory disease of the tooth gums.^32^ Other constituents such as methyl palmitate and 2-hexadecen-1-ol, 3,7,11,15-tetramethyl, which are common volatile components of most of the plant species, possess anti-inflammatory^33^ and other pharmacological activities. Hexadecanoic acid methyl ester (palmitic acid methyl ester) was also reported to inhibit phagocytic activity and nitric oxide production of certain cells and decrease levels of TNF-*α*, PGE_2_ and IL-10 without affecting ATP levels.^34–36^ It may be possible that either these identified compounds or some other unknown compounds or the synergistic effect of all these compounds together contribute to the brilliant anticancer activity against breast cancer as well as anti-inflammatory property of DBME.

The findings suggest that *D. burmannii* is a promising source of potential anti-inflammatory agents and may be useful as an anticancer agent against breast carcinoma. Flow cytometric and western blotting studies proposed that DBME induced cell cycle arrest and apoptosis in MCF-7 cells. Moreover, DBME also displayed a potent anti-inflammatory activity in murine macrophages by targeting the vital genes involved in inflammation. These outcomes from the study suggested that different pathways are involved in the anticancer activity of *D. burmannii*, especially targeting the inflammatory tumor microenvironment, and this activity can be attributed to the active components present in it. Although it is reasonable to correlate the identified compounds and their medicinal properties supported by the literature reports, this identification is based on the matching score and not the actual most of the times and it may be possible that there are several unknown compounds that are not present in any database that might also contribute to the bioactivity. Furthermore, we cannot neglect the possibility that GCMS can detect only volatile compounds in DBME. Therefore, we intend to expand our research toward the identification of the other unknown compounds in the active fractions for future studies. The crude extract is a mixture of several compounds and their biological activities are attributed to their nature and abundance. Therefore, it may be possible that some compounds are responsible for the anti-inflammatory activity against RAW 264.7 cells and some are responsible for anticancer activity against MCF-7 cells or these properties resulted from the synergism between the several compounds present in DBME.

## Materials and Methods

### Chemicals

Fetal bovine serum (FBS; US origin) was purchased from HyClone Laboratories Inc., South Logan, UT, USA. Anti-caspase-3, anti-caspase-8, anti-PARP, anti-BID, anti-Bax, anti-COX-2 and anti-beta-actin antibodies were purchased from OriGene Technologies, Inc., Rockville, MD, USA. Anti-p53, anti-Bcl-2 (NT), anti-caspase-9 and anti-iNOS antibodies were purchased from AnaSpec, Inc., Fremont, CA, USA. Anti-Cdc25C, anti-CDK1, anti-CDK2, anti-Cyclin A1 and anti-Cyclin B1 antibodies were purchased from Bioss, Inc., Woburn, MA, USA. Anti-p21 (WAF1, Cip1) was purchased from eBioscience, San Diego, CA, USA. Anti-cyclin D1 and anti-cdk-4 antibodies were purchased from Cell Signaling Technology, Inc., Danvers, MA, USA. Cell Proliferation Reagent WST-1 and the Annexin-V-FLUOS Kit were purchased from Roche Diagnostics, Mannheim, Germany. Alkaline phosphatase-conjugated anti-Rabbit secondary antibody was purchased from RockLand Immunochemicals Inc., Gilbertsville, PA, USA. Z-IETD-FMK (caspase-8 inhibitor) and Z-LEHD-FMK (caspase-9 inhibitor) were purchased from Abcam, Cambridge, MA, USA. All the other chemicals and solvents are of analytical or molecular biology grade and procured locally.

### Plant collection and extract preparation

A sample of the insectivorous plant *D. burmannii* Vahl. was collected in January 2014 from villages in the Bankura district in the state of West Bengal, India and authenticated by the Botanical Survey of India, Kolkata, India. The powdered shadow dried sample (100 g) was then stirred using a magnetic stirrer (Remi Sales & Engineering Ltd., Mumbai, India) with 70% methanol in water (1000 ml) for 15 h followed by centrifugation at 2850 *g*. The supernatant was decanted and the process was repeated by adding more solvent to the precipitated pellet. The supernatants obtained from the two phases were mixed and concentrated in a rotary evaporator (BÜCHI Labortechnik AG, Flawil, Switzerland) at 40 °C and then lyophilized and labeled as DBME.

### Cell lines and culture

Human lung adenocarcinoma (A549), human breast adenocarcinoma (MCF-7), human cervical carcinoma (HeLa), human hepatocellular carcinoma (HepG2), human glioblastoma (U87), murine macrophage (RAW 264.7) and human lung fibroblast (WI-38) cell lines were purchased from National Centre for Cell Science, Pune, India. All the cells were grown in DMEM except A549 cells, which were grown in Ham’s F-12 medium. Both the media were supplemented with 10% (v/v) FBS, 100 U/ml Penicillin G, 50 *μ*g/ml Gentamycin sulphate, 100 *μ*g/ml Streptomycin and 2.5 *μ*g/ml Amphotericin B. Cells were maintained in the laboratory at 37 °C in a humidified atmosphere containing 5% CO_2_ in CO_2_ incubator.

### WST-1 cytotoxicity assay

Cell proliferation and cell viability were quantified using the WST-1 Cell Proliferation Reagent (Roche Diagnostics), according to the previously described method.^37^ All the cells (1×10^4^ cells/well) were cultured for 48 h in 100 *μ*l/well medium containing DBME ranging from 0 to 200 *μ*g/ml in 96-well culture plate. At the end of the treatment, 10 μl/well of WST-1 reagent was added and the plate was incubated at 37 °C for an additional 2 h. Cell proliferation and viability were quantified by measuring absorbance at 460 nm using a microplate ELISA reader MULTISKAN EX (Thermo Electron Corporation, Waltham, MA, USA).

### Cell cycle analysis

Cell cycle analysis was performed by flow cytometry as previously described.^37^ In one experiment, MCF-7 cells (1×10^6^) were treated with DBME (0–200 *μ*g/ml) for 48 h, and in another experiment, cells were treated with DBME (200 *μ*g/ml) for different time intervals (0–48 h). At the end of the treatment, cells were harvested, fixed, treated with RNAse A and stained with propidium iodide, and the distribution of different cell cycle phases was determined on FACS Verse (Becton Dickinson, Franklin Lakes, NJ, USA) equipped with 488 nm (Blue), 405 nm (Violet) and 640 nm (Red) solid-state laser light. A total of 10 000 events were acquired, and data analysis was carried out using the FACSuite software Version 1.0.3.2942 (Becton Dickinson).

### Apoptosis versus necrosis

The identification of apoptotic and necrotic MCF-7 cells in DBME-treated samples was performed using the Annexin-V-FLUOS Staining Kit (Roche Diagnostics). At the end of the treatment, cells were harvested, thoroughly washed and labeled with PI and FITC according to the protocol described by the kit manufacturer. The distribution of differentially labeled cells was identified by flow cytometer. For the experiments that use inhibitors, cells were pretreated with 25 *μ*M caspase-8-specific inhibitor Z-IETD-fmk or 25 *μ*M caspase-9-specific inhibitor Z-LEHD-fmk or both, for 4 h before DBME treatment (200 *μ*g/ml) and assessment of apoptosis.

### Nitrite and TNF-*α* determination

RAW 264.7 cells were seeded at a density of 1×10^5^ cells/well in 24-well plates and then treated with DBME (30, 50, 80 *μ*g/ml) for 12 h. After pretreatment, cells were stimulated with or without LPS (1 *μ*g/ml) for 24 h. For nitrite determination, Griess reaction assay was used and presumed to reflect NO levels. Briefly, 300 *μ*l of cell culture medium was mixed with equal volume of Griess reagent (1 : 1 mixture of 1% (w/v) sulfanilamide in 5% (v/v) phosphoric acid and 0.1% (w/v) naphthyl ethylenediamine dihydrochloride), incubated at room temperature for 15 min and then the absorbance was measured at 540 nm. Fresh culture medium was used as the blank, and the amount of nitrite in the samples was measured with the sodium nitrite serial dilution standard curve. TNF-*α* levels in treated and untreated macrophage culture media were quantified using the EIA Kit (Amersham Tumor Necrosis Factor Alpha ((m)TNF-*α*) Mouse, Biotrak ELISA System, GE Healthcare, BUX, UK) according to the manufacturer’s instructions.

### Determination of intracellular ROS

The level of intracellular ROS was determined using dichlorodihydrofluorescein diacetate (DCF-DA) fluorescence. RAW 264.7 cells were seeded at a density of 2×10^5^ cells/well in 12-well plates and then treated with DBME (30, 50, 80 *μ*g/ml) for 12 h. After pretreatment, cells were stimulated with or without LPS (1 *μ*g/ml) for 24 h. The media was removed, and the cells were incubated with DCF-DA (20 *μ*M) dissolved in serum-free media for 30 min at 37 °C in dark. Cells were then harvested, washed and analyzed by flow cytometry. The fluorescent population of cells was selected, and mean fluorescence intensity was calculated using the FACSuite software Version 1.0.3.2942 (Becton Dickinson). The fluorescence intensity in the cell corresponds to the ROS generated inside the cell.

### Western blotting analysis

For the anticancer study, MCF-7 cells were treated with DBME (200 *μ*g/ml) for 6–48 h. For the anti-inflammatory study, RAW 264.7 cells were treated with DBME (30, 50, 80 *μ*g/ml) for 24 h. After treatment, cells were thoroughly washed with PBS and lysed with triple detergent cell lysis buffer (50 mM Tris-Cl, pH 8, 150 mM NaCl, 0.02% Sodium azide, 1% triton X-100, 0.1% sodium dodecyl sulphate, 0.5% sodium deoxycholate, 1 *μ*g/ml aprotinin, 100 *μ*g/ml phenyl-methyl-sulfonyl fluoride). The lysates were centrifuged at 13 800 *g* for 20 min at 4 °C, and the protein concentration was measured by a Folin–Lowry method. Equal amounts of proteins (40 *μ*g) were loaded in 12–15% SDS-PAGE. The resolved proteins were transferred to PVDF membrane and blocked with 5% non-fat dry milk in Tris-buffered saline. After blocking, the membranes were incubated with the corresponding primary antibodies separately overnight at 4 °C. The membranes were washed with TBS-T (0.01% of Tween-20 in TBS) and incubated with alkaline phosphatase-conjugated anti-Rabbit IgG antibody at room temperature in the dark for 3–4 h. The blots were developed with BCIP/NBT substrate, and the images were taken using the imaging system EC3 Chemi HR (UVP, Upland, CA, USA).

### RNA extraction and reverse transcriptase (RT)-PCR

Total cellular RNA from treated 1×10^7^ RAW 264.7 cells was isolated using the RNeasy Mini Kit, Qiagen (Hilden, Germany) following the manufacturer’s instructions. From each sample, 5 *μ*g of RNA was reverse-transcribed (RT) using M-MuLV RT using the RevertAid H-Minus First Strand cDNA Synthesis Kit, Thermo Fisher Scientific Inc. (Waltham, MA, USA). Then PCR analyses were performed on the aliquots of the cDNA preparations to detect iNOS, COX-2, TNF-*α* and *β*-actin (as an internal standard) gene expression using a thermal cycler (Veriti, Life Technologies Corporation, Carlsbad, CA, USA). The primers used for this study are provided in Table 3.

### Gas chromatography mass spectrometry (GCMS) analysis of DBME

To identify the probable chemical constituents in DBME (dissolved in methanol), GCMS was performed at South Indian Textile Research Association (SITRA), Coimbatore, India by Thermo GC—Trace Ultra Version: 5.0, Thermo MS DSQ II equipment in DB 35-MS capillary column (Standard Non-Polar) (Dimension: 30 m, ID: 0.25 mm, Film: 0.25 *μ*m) with initial oven temperature of 75 °C (hold time 2 min) and end temperature 260 °C (Ramp no-3). In all, 1 *μ*l of a sample was injected and run with Helium gas (flow rate 1 ml/min) in a splitless mode. The mass spectrometry was operated with vacuum pressure of 60 mTorr, transfer line temperatue 260 °C, ion trap temperature 200 °C and ionization energy of 70 ev. The chromatogram obtained from the GC was then analyzed by the mass spectrometry (MS) to get the mass of all the fractions. The major compounds in DBME were identified by comparing their retention times with those of authentic compounds, and the spectral data were obtained from the database of National Institute Standard and Technology (NIST) with a MS-library version 2011. The name, molecular weight, the percentage area and structure of the components in the DBME were determined.

### Statistical analysis

All spectrophotometric data were reported as the mean±S.D. of six measurements. Statistical analysis was performed by KyPlot version 2.0 beta 15 (32 bit). IC_50_ values were calculated by the formula, *Y*=100*A1/(*X*+*A*1), where *A*1=IC_50_, *Y*=response (*Y*=100% when *X*=0) and *X*=inhibitory concentration. The IC_50_ values were compared by paired *t*-test. *P*<0.05 was considered significant.

## Figures and Tables

**Table 1:** IC_50_ values of DBME against A549, MCF-7, HeLa, HepG2, U87 and WI-38 cells

*Cell line*	*IC_50_ (*μ*g/ml)*
A549 (lung)	1064.75±94.88
MCF-7 (breast)	120.94±1.91
HeLa (cervical)	1231.45±126.68
HepG2 (liver)	1577.07±447.89
U87 (brain)	1062.78±111.19
WI-38 (normal fibroblast)	1389.16±227.54

All the IC_50_ values are expressed as mean±S.D. (*N*=6).

**Table 2:** List of probable compounds present in DBME analyzed by GCMS

	*Name of probable compounds*	*Molecular formula*	*M.W.*	*r.t.*	*% area*	*Structure*
1	2,2,5-Trimethyl-cyclohexane-1,4-diol	C_9_H_18_O_2_	158	10.31	1.48	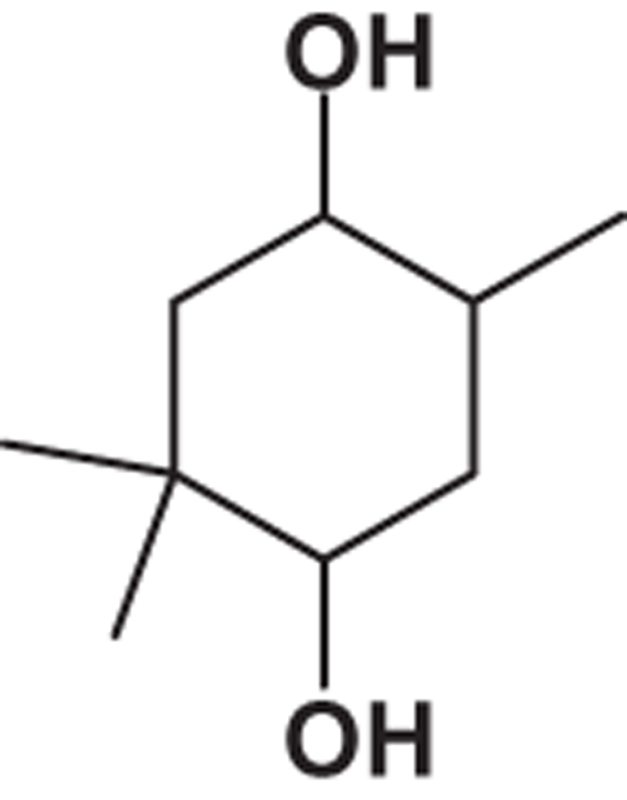
2	1-Tetradecanol	C_14_H_30_O	214	16.83	6.26	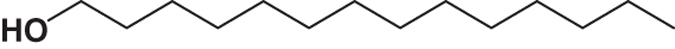
3	Tetradecanoic acid	C_14_H_28_O_2_	228	18.81	20.85	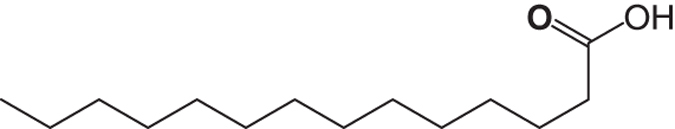
4	2,2-dimethyl-5-(3-methyloxiran-2-yl)cyclohexanone	C_11_H_18_O_2_	182	20.14	2.75	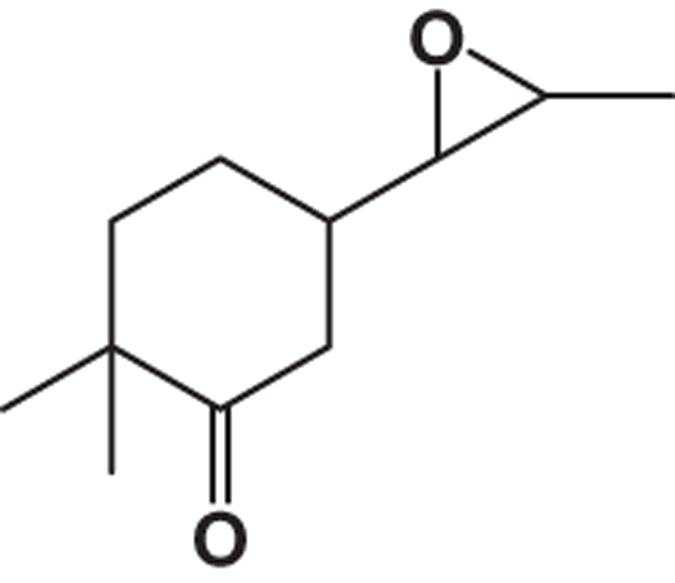
5	Hexadecen-1-ol, trans-9	C_16_H_32_O	240	21.07	14.90	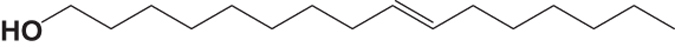
6	Hexadecanoic acid, methyl ester or methyl palmitate	C_17_H_34_O_2_	270	21.93	3.98	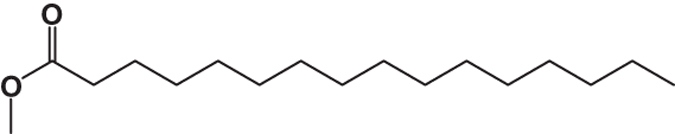
7	Hexadecanoic acid or palmitic acid	C_16_H_32_O_2_	256	22.89	24.19	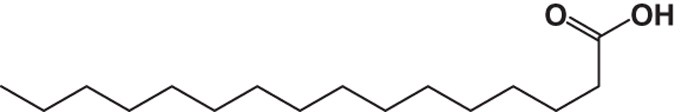
8	2-Hexadecen-1-ol, 3,7,11,15-tetramethyl	C_20_H_40_O	296	25.40	3.02	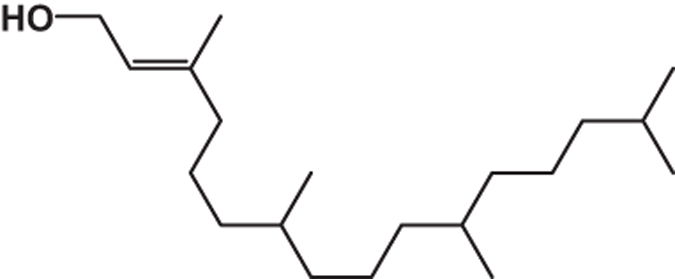
9	Octadecanoic acid or stearic acid	C_18_H_36_O_2_	284	26.78	3.11	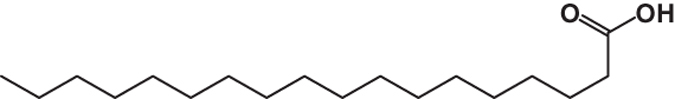
10	2-(hexadecyloxy) ethanol	C_18_H_38_O_2_	286	30.79	3.15	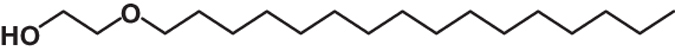
11	Beta-doradecin	C_40_H_52_O_3_	580	33.59	1.25	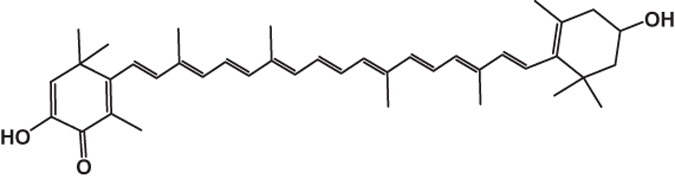
12	8,8-Dimethyl-3-(4-methyl-5-oxo-2,5-dihydrofuran-2-yloxymethylene)-3a,4,6,7,8,8b-hexahydro-3H-indeno[1,2-b]furan-2,5-dione	C_19_H_20_O_6_	344	38.28	1.26	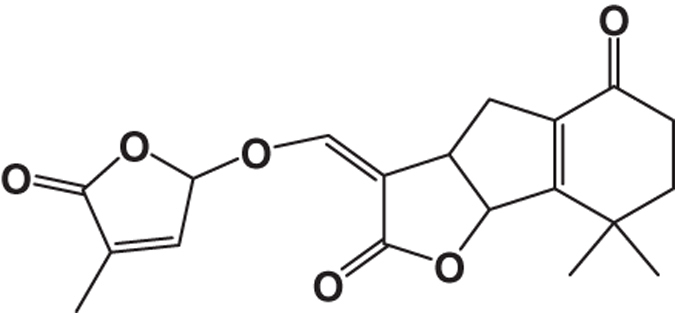

**Table 3:** List of the primers used for reverse transcriptase (RT)-PCR

*Gene studied*	*Forward primer*	*Reverse primer*
iNOS	5′-CCCTTCCGAAGTTTCTGGCAGC-3′	5′-AACGTAGACCTTGGGTTTTGCC-3′
COX-2	5′-GGAGAGACTATCAAGATAGT-3′	5′-ATGGTCAGTAGACTTTTACA-3′
TNF-*α*	5′-ATGAGCACAGAAAGCATGATC-3′	5′-TACAGGCTTGTCACTCGAATT-3′
*β*-Actin	5′-TCATGAAGTGTGACGTTGACATCCGT-3′	5′-CCTAGAAGCATTTGCGGTGCACGATG-3′

## References

[bib1] Mandal S , Hazra B , Sarkar R , Biswas S , Mandal N . Assessment of the antioxidant and reactive oxygen species scavenging activity of methanolic extract of *Caesalpinia crista* leaf. Evid Based Complement Altern Med 2011; 2011: 173768. 10.1093/ecam/nep072 PMC3136223 19596746

[bib2] Ferlay J , Soerjomataram I , Ervik M , Dikshit R , Eser S , Mathers C et al. GLOBOCAN 2012 1.0. International Agency for Research on Cancer, 2013. Available from: http://globocan.iarc.fr/.

[bib3] Li H , Fan X , Houghton JM . Tumor microenvironment: the role of the tumor stroma in cancer. J Cell Biochem 2007; 101: 805–815. 17226777 10.1002/jcb.21159

[bib4] Liu J , Wan J , He CW . Rationale for the use of natural anti-inflammatory agents in cancer chemotherapy. N Am J Med Sci 2010; 3: 160–166.

[bib5] Wiart C . Ethnopharmacology of Medicinal Plants: Asia and the Pacific. Humana press Inc.: Totowa, NJ, USA, 2007, pp 37–38.

[bib6] Hema B , Bhupendra S , Mohamed Saleem TS , Gauthaman K . Anticonvulsant effect of *Drosera burmannii* Vahl. Int J Appl Res Nat Prod 2009; 2: 1–4.

[bib7] Raju A , Christina AJM , Mayakrishnan A . Antitumor potential of ethanol and aqueous extracts of *Drosera burmannii* Vahl against Dalton’s ascitic lymphoma bearing mice. J Pharm Res 2012; 5: 1418–1423.

[bib8] Ghate NB , Chaudhuri C , Das A , Panja S , Mandal N . An antioxidant extract of the insectivorous plant *Drosera burmannii* Vahl. alleviates iron-induced oxidative stress and hepatic injury in mice. PLoS One 2015; 10: e0128221. 26010614 10.1371/journal.pone.0128221 PMC4444084

[bib9] Martinez V , Barber´a O , S´anchez-Parareda J , Marco JA . Phenolic and acetylenic metabolites from *Artemisia assoana*. Phytochemistry 1987; 26: 2619–2624.

[bib10] Torres K , Horwitz SB . Mechanisms of taxol-induced cell death are concentration dependent. Cancer Res 1998; 58: 3620–3626. 9721870

[bib11] Sun W , Wang W , Kim J , Keng P , Yang S , Zhang H et al. Anti-cancer effect of resveratrol is associated with induction of apoptosis via a mitochondrial pathway alignment. Adv Exp Med Biol 2008; 614: 179–186. 18290328 10.1007/978-0-387-74911-2_21

[bib12] Wang Y , Ji P , Liu J , Broaddus RR , Xue F , Zhang W . Centrosome-associated regulators of the G(2)/M checkpoint as targets for cancer therapy. Mol Cancer 2009; 8: 8. 19216791 10.1186/1476-4598-8-8 PMC2657106

[bib13] Murray AW . Recycling the cell cycle: cyclins revisited. Cell 2004; 116: 221–234. 14744433 10.1016/s0092-8674(03)01080-8

[bib14] Millar JB , Blevitt J , Gerace L , Sadhu K , Featherstone C , Russell P . p55CDC25 is a nuclear protein required for the initiation of mitosis in human cells. Proc Natl Acad Sci USA 1991; 88: 10500–10504. 1961714 10.1073/pnas.88.23.10500 PMC52956

[bib15] Agarwal M , Agarwal A , Taylor WR , Stark GR . p53 controls both the G2/M and the G1 cell cycle checkpoints and mediates reversible growth arrest in human fibroblasts. Proc Natl Acad Sci USA 1995; 92: 8493–8497. 7667317 10.1073/pnas.92.18.8493 PMC41183

[bib16] Taylor WR , Stark GR . Regulation of the G2/M transition by p53. Oncogene 2001; 20: 1803–1815. 11313928 10.1038/sj.onc.1204252

[bib17] Nicholson DW . Caspase structure, proteolytic substrates, and function during apoptotic cell death. Cell Death Differ 1999; 6: 1028–1042. 10578171 10.1038/sj.cdd.4400598

[bib18] Zou H , Li Y , Liu X , Wang X . An APAF-1.cytochrome C multimeric complex is a functional apoptosome that activates procaspase-9. J Biol Chem 1999; 274: 11549–11556. 10206961 10.1074/jbc.274.17.11549

[bib19] Pastorino JG , Chen ST , Tafani M , Snyder JW , Farber JL . The overexpression of Bax produces cell death upon induction of the mitochondrial permeability transition. J Biol Chem 1998; 273: 7770–7775. 9516487 10.1074/jbc.273.13.7770

[bib20] Li H , Zhu H , Xu CJ , Yuan J . Cleavage of BID by caspase 8 mediates the mitochondrial damage in the Fas pathway of apoptosis. Cell 1998; 94: 491–501. 9727492 10.1016/s0092-8674(00)81590-1

[bib21] Hasasna HE , Athamneh K , Samri HA , Karuvantevida N , Dhaheri YA , Hisaindee S et al. *Rhus coriaria* induces senescence and autophagic cell death in breast cancer cells through a mechanism involving p38 and ERK1/2 activation. Sci Rep 2015; 5: 13013. 26263881 10.1038/srep13013 PMC4532997

[bib22] Xue L , Fletcher GC , Tolkovsky AM . Mitochondria are selectively eliminated from eukaryotic cells after blockade of caspases during apoptosis. Curr Biol 2001; 11: 361–365. 11267874 10.1016/s0960-9822(01)00100-2

[bib23] Yu L , Alva A , Su H , Dutt P , Freundt E , Welsh S et al. Regulation of an ATG7-beclin 1 program of autophagic cell death by caspase-8. Science 2004; 304: 1500–1502. 15131264 10.1126/science.1096645

[bib24] Kostourou V , Cartwright JE , Johnstone AP , Boult JK , Cullis ER , Whitley G . The role of tumour-derived iNOS in tumour progression and angiogenesis. Br J Cancer 2011; 104: 83–90. 21139581 10.1038/sj.bjc.6606034 PMC3039789

[bib25] Lechner M , Lirk P , Rieder J . Inducible nitric oxide synthase (iNOS) in tumor biology: the two sides of the same coin. Semin Cancer Biol 2005; 15: 277–289. 15914026 10.1016/j.semcancer.2005.04.004

[bib26] Zweifel BS , Davis TW , Ornberg RL , Masferrer JL . Direct evidence for a role of cyclooxygenase 2-derived prostaglandin E2 in human head and neck xenograft tumors. Cancer Res 2002; 62: 6706–6711. 12438270

[bib27] Lejeune FJ . Clinical use of TNF revisited: improving penetration of anti-cancer agents by increasing vascular permeability. J Clin Invest 2002; 110: 433–435. 12189235 10.1172/JCI16493 PMC150423

[bib28] Balkwill F . Tumor necrosis factor or tumor promoting factor? Cytokine Growth Factor Rev 2002; 13: 135–141. 11900989 10.1016/s1359-6101(01)00020-x

[bib29] Shi J , Kakuda Y , Yeung D . Antioxidative properties of lycopene and other carotenoids from tomatoes: synergistic effects. Biofactors 2004; 21: 203–210. 15630198 10.1002/biof.552210141

[bib30] Aparna V , Dileep KV , Mandal PK , Karthe P , Sadasivan C , Haridas M . Anti-inflammatory property of n-hexadecanoic acid: structural evidence and kinetic assessment. Chem Biol Drug Des 2012; 80: 434–439. 22642495 10.1111/j.1747-0285.2012.01418.x

[bib31] Harada H , Yamashita U , Kurihara H , Fukushi E , Kawabata J , Kamei Y . Antitumor activity of palmitic acid found as a selective cytotoxic substance in a marine red alga. Anticancer Res 2002; 22: 2587–2590. 12529968

[bib32] Hasturk H , Jones VL , Andry C , Kantarci A . 1-Tetradecanol complex reduces progression of *Porphyromonas gingivalis*-induced experimental periodontitis in rabbits. J Periodontol 2007; 78: 924–932. 17470028 10.1902/jop.2007.060293

[bib33] Saeed NM , El-Demerdash E , Abdel-Rahman HM , Algandaby MM , Al-Abbasi FA , Abdel-Naim AB . Anti-inflammatory activity of methyl palmitate and ethyl palmitate indifferent experimental rat models. Toxicol Appl Pharmacol 2012; 264: 84–93. 22842335 10.1016/j.taap.2012.07.020

[bib34] Lin HW , Liu CZ , Cao D , Chen PY , Chen MF , Lin SZ et al. Endogenous methyl palmitate modulates nicotinic receptor-mediated transmission in the superior cervical ganglion. Proc Natl Acad Sci USA 2009; 105: 19526–19531. 10.1073/pnas.0810262105 PMC2596137 19057014

[bib35] Sarkar S , Khan MF , Kaphalia BS , Ansari GA . Methyl palmitate inhibits lipopolysaccharide-stimulated phagocytic activity of rat peritoneal macrophages. J Biochem Mol Toxicol 2006; 20: 302–308. 17163484 10.1002/jbt.20150

[bib36] Cai P , Kaphalia BS , Ansari GA . Methyl palmitate: inhibitor of phagocytosis in primary rat Kupffer cells. Toxicology 2005; 210: 197–204. 15840433 10.1016/j.tox.2005.02.001

[bib37] Ghate NB , Hazra B , Sarkar R , Mandal N . *In vitro* anticancer activity of *Spondias pinnata* bark on human lung and breast carcinoma. Cytotechnology 2013; 66: 209–218. 23686547 10.1007/s10616-013-9553-7 PMC3918266

